# The future of training in psychiatry in Europe

**DOI:** 10.1192/j.eurpsy.2024.112

**Published:** 2024-08-27

**Authors:** K. Krysta

**Affiliations:** Department of Rehabilitation Psychiatry, Medical University of Silesia, Katowice, Poland

## Abstract

**Abstract:**

Telepsychiatry has emerged as a transformative force in the field of mental health care, addressing disparities in service delivery and increasing access to care. This exploration focuses on the role of telepsychiatry in achieving equitable mental health care for individuals with intellectual disabilities (ID). Intellectual disabilities affect millions globally, posing significant public health challenges. This vulnerable population encounters numerous barriers in accessing quality mental health care, including geographical isolation, limited transportation options, and a shortage of specialized providers. Telepsychiatry offers a promising solution, leveraging technology to overcome these challenges. The presentation reviews the current landscape of mental health care for individuals with intellectual disabilities and the specific barriers they encounter. It highlights the potential benefits of telepsychiatry, including increased availability of specialized care, reduced geographical barriers, and enhanced caregiver support. Ethical considerations and best practices associated with implementing telepsychiatry in the context of intellectual disabilities are discussed. Case studies and success stories illustrate how telepsychiatry positively impacts individuals with intellectual disabilities and their families. In conclusion, telepsychiatry plays a promising role in promoting equitable mental health care for individuals with intellectual disabilities. Embracing technology and adopting best practices pave the way for a more inclusive and accessible mental health care system, leaving no one behind.

**Disclosure of Interest:**

None Declared

